# Intelligent Extraction of Minimum Burden in Medium-Length Hole Blasting Using Combined Region Growing and DBSCAN

**DOI:** 10.3390/s26103086

**Published:** 2026-05-13

**Authors:** Yu Bai, Yachun Mao, Shuai Zhen, Jing Liu, Shuo Fan

**Affiliations:** College of Resources and Civil Engineering, Northeastern University, Shenyang 110819, China; 2210436@stu.neu.edu.cn (Y.B.); 2390158@stu.neu.edu.cn (S.Z.); 2490129@stu.neu.edu.cn (S.F.)

**Keywords:** medium-length hole blasting, minimum burden, region-growing algorithm, DBSCAN, intelligent extraction of bench slope surfaces

## Abstract

**Highlights:**

A novel minimum burden extraction method is developed by combining region growing and DBSCAN optimisation, which mitigates over-segmentation and boundary loss and enhances slope surface extraction accuracy. An adaptive parameter strategy is proposed for point cloud processing, where DBSCAN and region-growing parameters are determined automatically to avoid manual subjectivity in complex mining environments. Automatic calculation of minimum burden is realised via 3D borehole modelling and the shortest Euclidean distance algorithm, overcoming the drawbacks of traditional manual measurement.

**What are the main findings?**

- The proposed integrated method of region growing and adaptive DBSCAN enables accurate intelligent extraction of the minimum burden in medium-length hole blasting, yielding a mean absolute error of 0.077 m and a mean relative error of 2.68% in field validation at the Huatailong open-pit mine.
- The method exhibits robust adaptability across diverse open-pit mining scenarios, effectively suppressing over-segmentation and noise interference from rock protrusions and blasting residues, and outperforms the single region-growing algorithm in both regular and complex slope conditions.

**What are the implications of the main findings?**

- This study breaks through the limitation that conventional manual methods cannot directly measure the minimum burden, providing a non-contact, automated, and high-precision technical approach for acquiring this core blasting parameter.
- The research provides a reliable quantitative basis for the optimisation of open-pit bench blasting design and fragmentation control, supporting the development of intelligent and digital blasting technology in mining engineering.

**Abstract:**

To address the difficulty of directly measuring the minimum burden in medium-length hole blasting and the low accuracy of single-algorithm extraction methods, this study proposes an automatic extraction method for the minimum burden based on combined region growing and DBSCAN. Using UAV-acquired three-dimensional point cloud data from open-pit mines, the elbow method is first applied to determine the clustering number of point cloud zenith distances, enabling initial extraction of the slope surface under roughness constraints. Subsequently, DBSCAN parameters are adaptively determined using the K-nearest neighbor average distance method, and density optimization is performed on the region-growing results to remove noise points such as rock protrusions and blasting residues, thereby refining the reconstruction of the free surface. Based on the reconstructed surface, the minimum burden is calculated using three-dimensional borehole modeling combined with the shortest Euclidean distance algorithm. Field experiments were conducted at the 5015 platform of the Huatailong open-pit mine in Tibet, with additional validation at the Qianshan limestone mine in Liaoyang and the Qidashan iron mine in Anshan. Results show that the proposed method effectively identifies slope free surfaces and accurately extracts the minimum burden. In the Huatailong case, the average absolute error was 0.077 m and the average relative error was 2.68%. The method provides a reliable basis for blasting fragmentation control and blast-hole pattern design in open-pit mines.

## 1. Introduction

In open-pit bench blasting engineering, the minimum burden, as a pivotal parameter controlling the direction of explosive energy release, plays a decisive role in determining blast fragmentation distribution, fly-rock distance, and construction safety. Its accurate calculation provides fundamental technical support for optimizing blast design and reducing mining costs [1,2]. As the foundational step for minimum burden extraction, modeling the spatial relationship between bench slope surfaces and blast holes in open-pit mines requires high-precision topographic feature extraction to achieve accurate matching between the free face and charge structure, thereby providing a quantitative basis for the optimization of blasting parameters [3,4]. The conventional manual measurement method for determining the minimum burden cannot directly acquire target data, relying instead on indirect approaches. This method suffers from insufficient accuracy and fails to meet the demands of modern mines for both blasting efficiency and safety performance [5,6,7,8]. Consequently, there is an urgent need for an automated extraction method based on 3D point cloud technology to provide a reliable data benchmark for the minimum burden in medium-length hole bench blasting design. In recent years, with the rapid development of digital mining technologies, a number of advanced approaches have been introduced to improve the acquisition and analysis of blasting-related geometric parameters [9]. UAV photogrammetry has been widely used for large-scale terrain reconstruction due to its flexibility and high efficiency, enabling rapid generation of high-resolution point cloud models of bench slopes. Meanwhile, LiDAR and terrestrial laser scanning technologies provide high-precision geometric data for complex rock surfaces, offering reliable support for accurate free-surface characterization. In addition, recent studies have explored blast-hole geometry reconstruction and digital blasting design frameworks to enhance the integration of spatial data and blasting parameter optimization. For instance, Fan et al. [10] proposed a novel intelligent framework for open-pit mining environment segmentation, demonstrating the potential of advanced data-driven methods in supporting digital mining workflows. Despite these advances, existing studies still lack a robust and automated approach specifically tailored for accurate minimum burden extraction under complex open-pit conditions.

Current methods for minimum burden extraction are primarily categorized into manual empirical approaches, such as visual interpretation based on experience, and automated methods based on point cloud segmentation. The former, while low in operational complexity, suffers from inherent limitations: it cannot directly extract the minimum burden and must rely on indirect estimation. Furthermore, it is constrained by issues such as time-consuming measurements for individual blast holes and safety hazards associated with operations on high and steep slopes, making it difficult to adapt to complex mining scenarios [11,12,13]. The latter category, leveraging its automation advantage, has become a research focus. Among these, the region-growing algorithm is widely applied in this field due to its ability to achieve slope surface extraction by leveraging constraints of topographic continuity [14,15]. For instance, Liu et al. [16] achieved high-precision extraction of loess plateau landforms by refining the growth criteria, reporting an accuracy of 96.9% with errors controlled within 10 m. Miyazaki et al. [17] employed a line-constrained region-growing method to construct road plane models, further validating the algorithm’s effectiveness in structured terrains. However, in mining scenarios, prevalent noise points—such as rock protrusions and blasting remnants—often induce over-segmentation in the region-growing algorithm. This, in turn, introduces deviations in free face extraction, thereby limiting its application for minimum burden extraction in open-pit mines. In recent years, intelligent recognition algorithms for complex scenarios have also provided valuable references for point cloud processing in mining environments. For instance, Xing et al. [18] proposed a lightweight object detection model for unstructured indoor scenes in home rehabilitation, which improves the stability of target recognition in complex environments through joint attention and prior knowledge. This indicates that in scenarios with significant noise and irregular object boundaries, relying solely on a single feature is often insufficient to obtain reliable results. Meanwhile, Xing et al. [19] developed a three-dimensional graph deep learning method for non-contact gesture recognition, which incorporates spatial structural relationships into the process of human motion recognition, demonstrating the importance of three-dimensional spatial features for complex object identification. Open-pit mine slope point clouds exhibit similar characteristics, including high noise levels, irregular boundaries, and complex spatial structures. Therefore, in the extraction of the minimum burden, it is also necessary to comprehensively utilize geometric continuity, density distribution, and boundary constraints to improve the accuracy of free-surface recognition.

Clustering algorithms offer a viable alternative to address the issue of noise interference. Among them, the DBSCAN algorithm demonstrates unique advantages in complex scenarios without predefining the number of clusters, as it can automatically eliminate noise points through density threshold control [20,21]. The original DBSCAN algorithm, introduced by Ester et al. [22], utilizes the ε-neighborhood and minimum points parameters to achieve cluster formation, providing a foundational framework for point cloud segmentation. Subsequent researchers, such as Li et al. [23], addressed the issue of missing point cloud data in mining scenarios by proposing an enhanced DBSCAN algorithm integrated with point cloud completion techniques. This improved method optimizes the clustering threshold through a Density Distribution Map (DDM) mechanism, significantly boosting the accuracy of structural plane extraction in hard rock pillars. However, when applied in isolation, the clustering performance of the DBSCAN algorithm is highly dependent on the configuration of its ε-value and minimum cluster size parameters. This dependency often leads to under-segmentation in areas with non-uniform point cloud density, such as the transitional zones between bench crests and toe areas in mining scenes. Furthermore, while existing research has largely focused on parameter self-adaptation—for instance, the method by Mohammad et al. [24] dynamically adjusts the neighborhood parameter using a field-of-view partitioning strategy, achieving local adaptability of the ε-value based on point cloud distance and density features, thereby enhancing clustering for non-uniformly distributed point clouds—such approaches still struggle to strike an effective balance between noise suppression and the preservation of free-surface boundary integrity.

Therefore, the combined application of the region-growing and DBSCAN algorithms provides a feasible solution to the aforementioned technical challenges. The region-growing algorithm relies on geometric continuity constraints, such as the zenith distance range and planarity of slope surfaces, enabling it to preferentially preserve the complete boundary characteristics of open-pit bench slopes. Meanwhile, the DBSCAN algorithm, through its density-threshold-based noise filtering capability, can effectively remove pseudo-slope point cloud clusters generated during the region-growing process due to rock protrusions and blasting residues, thereby mitigating the over-segmentation problem. Based on this, this study proposes an intelligent extraction method for the minimum burden in medium-length hole blasting using combined region growing and DBSCAN. Compared with existing approaches, the main innovations of the proposed method are summarized as follows: (1) A slope surface extraction method based on the complementarity between geometric continuity and density characteristics is proposed. The region-growing algorithm is first employed to preserve the complete boundary of the slope surface, with initial slope regions extracted using zenith angle clustering and roughness constraints. Subsequently, the DBSCAN algorithm is applied to eliminate low-density noise points such as rock protrusions and blasting residues. This strategy effectively overcomes the over-segmentation issue of region growing and the boundary loss problem of standalone DBSCAN. (2) An adaptive parameter determination framework for point cloud processing is established. The neighborhood scale for normal vector estimation is automatically determined based on spatial point cloud density, while the DBSCAN neighborhood radius is derived from the statistical characteristics of K-nearest neighbor distances. In addition, the minimum cluster size is adaptively computed according to the spatial density of slope point clouds. This approach avoids the subjectivity of manual parameter tuning and improves robustness across different mining environments. (3) An automated workflow for minimum burden calculation is developed. A three-dimensional borehole model is constructed to determine the geometric center of the explosive charge, and the shortest Euclidean distance from this center to the optimized slope surface is calculated. This enables direct and automated measurement of the minimum burden in medium-length hole blasting, providing a quantitative basis for blast design.

## 2. Method

**Figure 1:**
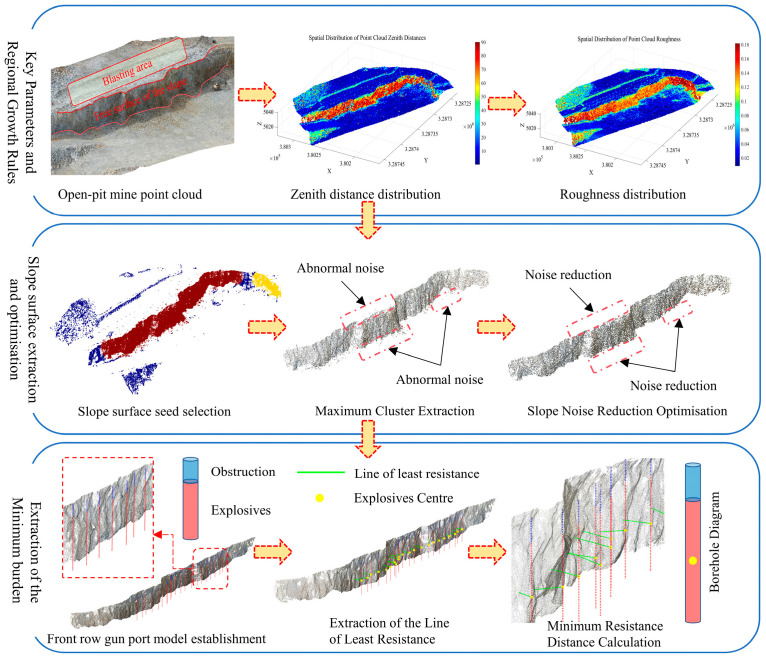
Workflow of the proposed method.

Figure 1 illustrates the workflow of the proposed method. The process mainly consists of the following stages: Key Parameters and Regional Growth Rules, Slope Surface Extraction and Optimisation, and Extraction of the Minimum burden.

### 2.1. Point Cloud Preprocessing

The raw point cloud obtained through UAV photogrammetry can effectively capture the spatial morphology of open-pit mine slopes. However, it is characterized by high point density and large data volume, and often contains non-geological objects such as workers, pickup trucks, and excavators. These factors not only increase the computational complexity of slope surface extraction but may also interfere with the identification of geometric features of the slope. Therefore, prior to slope surface extraction, the point cloud must be downsampled, and non-geological objects are removed using a progressive morphological filtering method.

(1) Raw Data Downsampling

**Figure 2:**
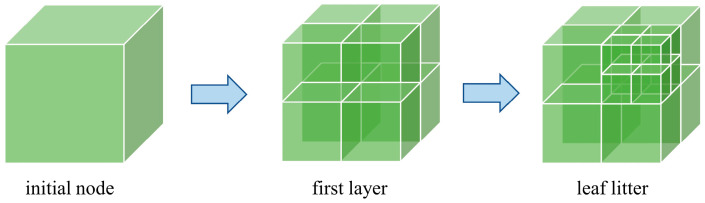
Principle of Octree-based dimensionality reduction.

During large-scale point cloud processing, the high-density characteristics of raw data can represent terrain details comprehensively. However, the large volume of redundant point cloud data significantly increases the computational burden of extracting open-pit slope surfaces. To address this issue, an Octree-based downsampling algorithm is adopted in the point cloud preprocessing stage to perform spatial dimensionality reduction on the raw data [25]. In this study, the minimum voxel edge length of the Octree structure is set to 0.08 m, and the voxel is further subdivided when the number of points within a voxel exceeds 10, as illustrated in Figure 2.

Let the spatial bounding box of the point cloud be defined as:

(1) $$B=\left(x_{\min},x_{\max},y_{\min},y_{\max},z_{\min},z_{\max}\right)$$

When the number of points within a voxel exceeds the threshold *Nv*, the voxel is further subdivided. Otherwise, the centroid of the voxel is retained as the representative point. The coordinates of the voxel centroid are calculated as

(2) $$P_{c}=\left(\frac{1}{n}\sum_{i=1}^{n}x_{i},\frac{1}{n}\sum_{i=1}^{n}y_{i},\frac{1}{n}\sum_{i=1}^{n}z_{i},\right)$$

where *n* denotes the number of points contained in the voxel.

(2) Removal of Non-Geological Objects

**Figure 3:**
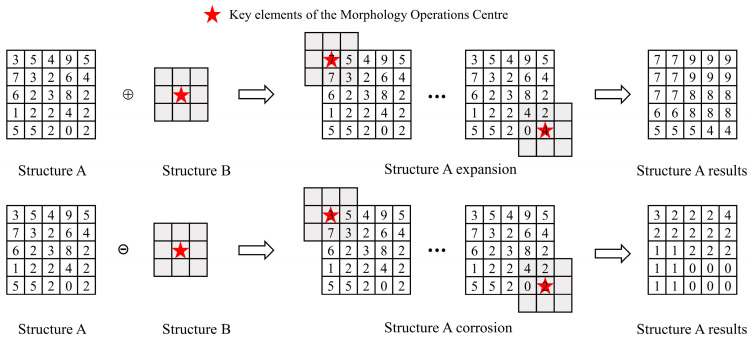
Illustration of the dilation–erosion principle.

Non-geological objects in open-pit mine point clouds, such as workers, pickup trucks, and excavators, can interfere with slope surface extraction. In this study, a Progressive Morphological Filter (PMF) is adopted to remove non-geological objects [26]. This method exploits the spatial continuity of elevation on geological slope surfaces and the abrupt elevation changes associated with non-geological objects. By applying morphological dilation and erosion operations, the two types of points can be effectively distinguished. Taking a randomly generated elevation sequence as an example, the principle of dilation–erosion operations is illustrated in Figure 3.

Core equation of morphological operations

(3) $$Z_{g,k}(x,y)=\min\left\{Z(x,y),Z_{g,k-1}(x,y)+d_{k}\right\}$$

where *Z*(*x*, *y*) represents the elevation value of the original point cloud at coordinate (*x*, *y*); $Z_{g,k}(x,y)$ denotes the estimated ground elevation after the *k*-th iteration;

By progressively increasing the size of the structural element, small-scale elements first remove objects such as workers and pickup trucks, while larger-scale elements subsequently eliminate larger objects such as excavators. Finally, the elevation difference between the point cloud and the estimated ground surface is evaluated. Points with differences exceeding the predefined threshold are identified as non-geological objects and removed, while geological target points are retained.

### 2.2. Key Parameters and Region-Growing Rules

From the perspective of point cloud segmentation, the region-growing method relies on normal consistency and surface continuity, which can effectively preserve the overall connectivity of the slope surface, but is sensitive to discrete noise. DBSCAN, on the other hand, partitions data based on point density, which can effectively remove low-density outliers, but provides weak constraints on geometric boundaries. Open-pit mine point clouds exhibit both structural continuity and dispersed noise, making it difficult for a single method to address both aspects simultaneously. By taking the initial slope surface obtained from region growing as the basis and then applying DBSCAN to filter local density, it is possible to reduce noise interference while preserving the overall morphology of the slope, resulting in more spatially stable outcomes with clearer boundaries.

(1) Zenith angle

The zenith angle is a key parameter used to describe the spatial orientation of point cloud normal vectors. It is defined as the complementary angle between the point cloud normal vector and the positive direction of the *Z*-axis in the Cartesian coordinate system. Due to the relatively stable inclination characteristics of open-pit bench slopes, the distribution of zenith angles on slope surfaces typically exhibits clustering behavior, whereas the zenith angles in non-slope regions are more scattered. Based on this characteristic, an initial separation between slope-surface point clouds and non-slope point clouds can be achieved.

In this study, the seed range of the zenith angle is determined according to the slope-surface cluster angles obtained using the elbow method [27]. The calculation procedure of the zenith angle is described as follows.

The normal vector of each point is estimated using the K-nearest neighbors (KNN) algorithm. Suppose the normal vector of a point in the point cloud is $\vec{n}=\left(n_{x},n_{y},n_{z}\right)$, where *n*_*z*_ represents the component of the normal vector along the *Z*-axis. The zenith angle *θ* of the point is calculated as

(4) $$\theta =\arccos\left(\left|\vec{n}\right.\cdot \left.{\vec{e}}_{z}\right|\right)\times \frac{180{}^{\circ}}{\pi }$$

where ${\vec{e}}_{z}=\left(0,0,1\right)$ denotes the unit vector in the positive direction of the *Z*-axis, and arccos() represents the inverse cosine function with a value range of $\left[0{}^{\circ},90{}^{\circ}\right]$.

To improve the adaptability of the proposed method under different point cloud density conditions, an adaptive neighborhood-scale determination strategy is adopted for normal vector estimation. Let *N* denote the total number of points in the point cloud and *A* represent the area of the study region. The average point cloud density can therefore be expressed as:

(5) $$\rho =\frac{N}{A}$$

Based on the spatial distribution characteristics of the point cloud, the number of neighboring points *K* can be defined as

(6) $$K=\rho \pi r^{2}$$

where *r* denotes the neighborhood search radius, defined as $r=c\sqrt{A/N}$, with *c* = 2.5, and *K* is rounded up to the nearest integer. Through this strategy, the neighborhood scale used for normal vector estimation can be automatically determined according to the point cloud density, thereby avoiding the mismatch problem caused by using a fixed neighborhood scale for datasets from different mining areas.

After obtaining the zenith angle data, clustering analysis is performed on the zenith angle distribution using the elbow method. The optimal number of clusters is determined based on the variation trend of the within-cluster sum of squares (WCSS), thereby identifying the effective zenith angle interval of the slope surface $\left[\theta_{\mathrm{slope},\min},\theta_{\mathrm{slope},\max}\right]$, which serves as the primary constraint for selecting slope surface seed points.

(2) Roughness

The surface of open-pit bench slopes generally exhibits good micro-scale smoothness and relatively low roughness values. In contrast, non-slope regions, such as rock protrusions and blasting residues, show pronounced surface undulations, resulting in significantly higher roughness values. Based on this characteristic, potential slope-surface seed points can be further identified.

The K-nearest neighbors (KNN) method is adopted to calculate the roughness of each point. For a point *P*_*i*_ in the point cloud, its *K* neighboring points $\left\{P_{i1},P_{i2},\ldots ,P_{iK}\right\}$ are selected, and the elevation values of these neighboring points $\left\{z_{i1},z_{i2},\ldots ,z_{iK}\right\}$ are extracted. The roughness *R*_*i*_ of the point is defined as the standard deviation of the elevations of the neighboring points:

(7) $$R_{i}=\sqrt{\frac{1}{K-1}\sum_{j=1}^{K}{\left(z_{ij}-{\bar{z}}_{i}\right)}^{2}}$$

where ${\bar{z}}_{i}=\frac{1}{K}\sum_{j=1}^{K}z_{ij}$ represents the mean elevation value of the neighboring points, and *K* denotes the number of neighboring points.

To reduce the influence of empirically selected thresholds, the roughness screening threshold is adaptively determined according to the roughness distribution of slope-surface point clouds. Let *μ*_*R*_ denote the mean roughness of the initial slope-surface candidate points and *σ*_*R*_ denote the corresponding standard deviation. The roughness threshold is therefore defined as

(8) $$R_{th}=\mu_{R}+\sigma_{R}$$

When the point cloud roughness satisfies *R*_*i*_ ≤ *R*_*th*_, the point is identified as a potential slope-surface point.

The roughness threshold is determined jointly by the mean and standard deviation, which is derived from the overall distribution characteristics of point cloud roughness. In open-pit slope point clouds, the majority of points are concentrated on continuous slope surfaces, where roughness variation is relatively stable, while anomalous points such as rock protrusions and blasting residues are fewer in number and spatially dispersed, thus having limited influence on the overall statistical characteristics. On this basis, the mean reflects the central tendency of slope roughness, while the standard deviation describes local fluctuation intensity, forming a filtering range that adapts to data variation. Since anomalous points have already been partially suppressed through zenith angle constraints and the initial growth process, the data entering statistical computation mainly consist of slope surface points. Therefore, this threshold can stably reflect variations in slope surface characteristics and exhibits good adaptability under different point cloud densities and noise conditions.

(3) Region-Growing Criteria

The zenith angle constraint is prioritized. After clustering the zenith angles of the open-pit mine bench slope surfaces using the optimal number of clusters determined by the elbow method, the zenith angle range for the identified slope surface cluster is defined as $\left[\theta_{\mathrm{slope},\min},\theta_{\mathrm{slope},\max}\right]$, which reflects the stability of the overall inclination attitude of the slope. For any candidate point $P_{i}$, its zenith angle $\theta_{i}$ must satisfy the following condition:

(9) $$\theta_{\mathrm{slope},\min}\leq \theta_{i}\leq \theta_{\mathrm{slope},\max}$$

where $\theta_{i}$ is calculated from the point cloud normal vector according to Equation (4).

Secondly, a consistency constraint on roughness is applied to candidate points. Let the roughness of seed point $P_{s}$ be $R_{s}$, and the roughness of candidate point $P_{i}$ be *R_i_*. The following condition must be satisfied:

(10) $$|R_{i}-R_{s}|\leq \Delta R_{th}$$

The roughness difference threshold Δ*R_h_* is adaptively determined according to the statistical characteristics of roughness:

(11) $$\Delta R_{th}=\sigma_{R}$$

Finally, constraints are imposed on the normal vectors of the seed points. Since the slope surface can be approximated as a spatially continuous surface, the directions of the normal vectors of neighboring points should remain consistent to prevent the growth process from crossing slope boundaries. Let ${\vec{n}}_{s}$ denote the normal vector of the seed point *P*_*s*_, and ${\vec{n}}_{s}$ denote the normal vector of the candidate growth point *P*_*i*_. The angle between the two vectors, *φ*_*i*,*s*_, should satisfy

(12) $$\varphi_{i,s}=\arccos\left(\frac{\left|{\vec{n}}_{i}\cdot {\vec{n}}_{s}\right|}{\left\|{\vec{n}}_{i}\right\|\cdot \left\|{\vec{n}}_{s}\right\|}\right)\times {\frac{180}{\pi }}^{\circ }\leq \varphi_{th}$$

where $\varphi_{th}$ is the normal vector angle threshold, and ||⋅|| denotes the L2-norm of the vector; this constraint ensures spatial continuity of the growing cluster by controlling the deviation in normal vector directions, thereby preventing over-segmentation caused by local protrusions.

Based on the aforementioned constraints, the region-growing algorithm is implemented as follows: initial seed points are incorporated into a growth queue. Points are sequentially retrieved from the queue as current seeds, and their K-nearest neighbors are traversed. Candidate points satisfying the zenith angle constraint, roughness consistency constraint, and normal-vector consistency constraint specified in Equations (9)–(12) are added to the queue and simultaneously marked as grown. This process is iterated until the queue is emptied, ultimately yielding M discrete initial slope surface clusters $\left\{C_{1},C_{2},\ldots ,C_{M}\right\}$.

### 2.3. Slope Surface Extraction and Optimization

The region-growing algorithm extracts slope surfaces based on geometric continuity, which can fully preserve boundary features of the slope, but is sensitive to local low-density noise and prone to over-segmentation. The DBSCAN algorithm identifies noise based on point cloud density, effectively removing discrete outliers, but when used alone may lead to the loss of fine boundary details. Therefore, this study adopts a processing sequence in which region growing is first used to extract the initial slope surface, followed by DBSCAN-based density optimization, thereby combining the advantages of both methods to achieve accurate slope surface extraction. The initial slope clusters obtained by region growing may still contain pseudo-slope clusters and discrete noise points, and thus further filtering and optimization are required to improve extraction accuracy.

(1) Slope-Surface Cluster Filtering

Pseudo slope-surface clusters typically contain fewer points and exhibit a dispersed spatial distribution. Therefore, preliminary filtering can be performed based on the size characteristics of each point cloud cluster. Let $\left|C_{m}\right|$ denote the number of points in the *m*-th initial slope-surface cluster. The minimum cluster size threshold *N*_*min*_ is defined as

(13) $$N_{\min}=\mu_{c}-\sigma_{c}$$

where *μ*_*c*_ represents the mean number of points in all initial slope-surface clusters and $\sigma_{c}$ denotes the corresponding standard deviation. When a cluster satisfies $|C_{m}|<N_{\min}$, it is identified as a pseudo slope-surface cluster and removed. The remaining clusters are retained as candidate slope surfaces for subsequent density optimization.

(2) DBSCAN Density Optimization

After removing small-scale pseudo slope-surface clusters, the remaining slope-surface point clouds may still contain isolated noise points caused by rock protrusions and blasting residues. To address this issue, the DBSCAN algorithm is employed to perform density optimization on the slope-surface point clouds.

The key parameters of the DBSCAN algorithm include the neighborhood radius *ε* and the minimum cluster size MinPts [28]. The neighborhood radius *ε* is determined using the K-nearest neighbor average distance method. Specifically, *N*_*sample*_ points are randomly selected from the slope-surface clusters, and the average distance to the *P*_*k*_-nearest neighbors of each sample point, denoted as *d*_*k*_, are calculated and defined as follows:

(14) $$d_{k}=\frac{1}{K}\sum_{j=1}^{K}\|P_{k}-P_{kj}\|$$

where *P*_*k*_ denotes the *j*-th nearest neighbor of point *P*_*i*_, and *K* represents the number of neighboring points.

Based on this, the neighborhood radius *ε* is defined as the sum of the mean *μ*_*d*_ and the standard deviation *σ*_*d*_ of the average nearest-neighbor distances of the sampled points:

(15) $$\varepsilon =\mu_{d}+\sigma_{d}$$

The minimum cluster size MinPts is adaptively determined according to the spatial density of the slope-surface point cloud. The average spatial density of the slope-surface points *ρ*_*c*_ is defined as

(16) $$\rho_{C}=\frac{N_{C}}{V_{bbox}}$$

where *N*_*c*_ denotes the total number of slope-surface points involved in the DBSCAN clustering, and *V*_*bbox*_ represents the volume of the corresponding point cloud bounding box.

Let the volume of the *ε*-neighborhood sphere be

(17) $$V_{\varepsilon }=\frac{4}{3}\pi \varepsilon^{3}$$

The minimum cluster size MinPts is therefore defined as

(18) $$\mathrm{MinPts}=\max(5,\left[\rho_{C}\cdot V_{\varepsilon }\right])$$

where $\left[\cdot \right]$ denotes the ceiling operation, and *ρ*_*c*_⋅*V*_*ε*_ represents the theoretical expected number of points within the *ε*-neighborhood. To prevent excessively small thresholds caused by locally sparse regions, 5 is set as the minimum lower bound.

After determining the DBSCAN parameters using Equations (14)–(18), density-based clustering is performed on the slope-surface point cloud to extract the largest continuous slope-surface cluster while removing isolated noise points.

(3) Crest and Toe Line Constraint Optimization

To eliminate interferences such as redundant points on the crest platform and accumulated materials at the toe within the largest cluster after DBSCAN density optimization, and to accurately define the slope surface extent, constraints based on crest and toe lines are introduced. Firstly, leveraging the zenith angle range $\left[\theta_{\mathrm{slope},\min},\theta_{\mathrm{slope},\max}\right]$ of the slope surface determined by the elbow method, a point cloud subset *S*_subset_ conforming to this range is filtered from the largest cluster. Subsequently, *S*_subset_ is projected onto the X-Y plane, and a grayscale image is constructed using elevation values as intensities. The crest line $L_{\mathrm{crest}}$ and the toe line $L_{\mathrm{toe}}$ are extracted via the Canny operator. Finally, candidate seed points *P*(*x*,*y*,*z*) must simultaneously satisfy the condition of lying within the vertical region bounded by these two lines and meeting the zenith angle requirement. The feasible domain is defined as follows:

(19) $$P\in \Omega_{\mathrm{seed}}\Leftrightarrow \left\{\begin{array}{c} X_{\mathrm{toe},\min}\leq x\leq X_{\mathrm{crest},\max},Y_{\mathrm{toe},\min}\leq y\leq Y_{\mathrm{crest},\max}\\ Z_{\mathrm{toe},\min}\leq z\leq Z_{\mathrm{crest},\max},\theta_{P}\in [\theta_{\mathrm{slope},\min},\theta_{\mathrm{slope},\max}] \end{array}\right.$$

where $\Omega_{\mathrm{seed}}$ represents the feasible domain for seed points.

### 2.4. Minimum Burden Extraction

The minimum burden is defined as the shortest spatial distance from the equivalent center of explosive energy release to the slope surface. First, a 3D cylindrical blast hole model is constructed based on the coordinates of the blast hole top in the point cloud data, along with design parameters such as the blast hole radius and depth. Then, the geometric center $O(x_{O},y_{O},z_{O})$ of the explosive column is determined using the elevations of its top and bottom. Finally, the shortest distance from this center to the slope surface is calculated. Here, the elevation of the center is taken as the average of the top and bottom of the explosive column, while its planar coordinates coincide with the blast hole axis. The specific calculation method is as follows: taking all points $\left\{S_{k}(x_{k},y_{k},z_{k})\right\}$ on the free surface S as candidates, the Euclidean distance from the explosive center O to each point is computed. It should be noted that in this study, the extracted free surface is represented directly as a discrete point set rather than a fitted or triangulated surface. No surface reconstruction techniques, such as mesh generation or plane fitting, are applied. The minimum burden is therefore calculated based on point-to-point Euclidean distance, which avoids additional modeling errors introduced by surface fitting and preserves the original geometric characteristics of the point cloud. The minimum value among these distances is taken as the minimum burden *W*, expressed by the formula:

(20) $$W=\min\left\{\sqrt{{\left(x_{O}-x_{k}\right)}^{2}+{\left(y_{O}-y_{k}\right)}^{2}+{\left(z_{O}-z_{k}\right)}^{2}}\right\}\;(k=1,2,\ldots ,N_{S})$$

where $N_{S}$ denotes the total number of free surface points, and $x_{O},y_{O},z_{O}$ represent the *x*, *y*, *z* coordinates of the center of a given blast hole, respectively.

## 3. Experiments

### 3.1. Experimental Environment and Data Source

The experimental data were collected in September 2025, with the study area located on the 5015 platform of the Huatailong open-pit mine in Tibet, China. This area represents a typical open-pit bench blasting operation zone, where the slope morphology is relatively intact and therefore exhibits strong representativeness for experimental analysis. The data acquisition was conducted using a DJI M300 RTK unmanned aerial vehicle (UAV) equipped with a Zenmuse P1 full-frame aerial survey camera (both manufactured by SZ DJI Technology Co., Ltd., Shenzhen, China). Combined with multi-frequency GNSS positioning and intelligent flight path planning technology, three-dimensional point cloud data of the study area were obtained. According to the equipment specifications, the generated model achieves a planimetric accuracy of approximately 3 cm and a vertical accuracy of approximately 5 cm. The study area extends approximately 95.34 m in the transverse direction and 236.50 m in the longitudinal direction, with the raw point cloud containing approximately 10.36 million points.

### 3.2. Data Preprocessing and Experimental Environment

To reduce the impact of high-density point cloud data on computational efficiency and eliminate the interference of non-geological objects in slope surface recognition, the acquired raw point cloud data were first preprocessed. The preprocessing procedure mainly consists of two steps: point cloud downsampling and non-geological object removal.

**Figure 4:**
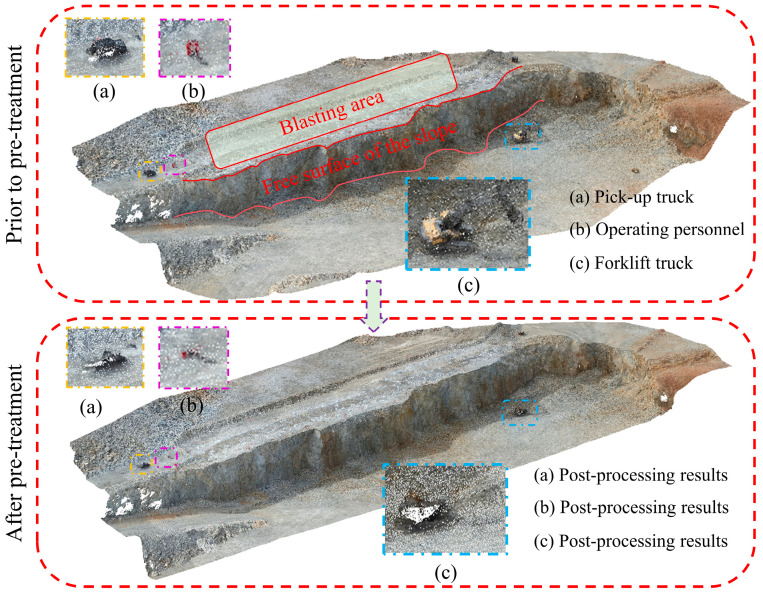
Point Cloud Information: Before and After Preprocessing.

During the downsampling stage, an Octree-based downsampling algorithm was employed to perform spatial dimensionality reduction on the raw point cloud. This process reduces data redundancy while preserving the overall structural characteristics of the terrain, thereby improving the computational efficiency of subsequent algorithms. Subsequently, to eliminate the influence of non-geological objects commonly present in open-pit mining environments—such as workers, pickup trucks, and construction machinery—on slope surface identification, a Progressive Morphological Filter (PMF) was applied to the point cloud data. This method enables the automatic identification and removal of non-geological point cloud objects. After preprocessing, the original point cloud containing approximately 10.36 million points was effectively reduced to 2,491,009 points, significantly decreasing the dataset size while retaining the primary geometric features of the slope surface. The experimental algorithms were implemented in MATLAB (version R2024b, MathWorks, Natick, MA, USA) and executed on a Windows 11 operating system. The computing platform was equipped with an Intel Core i7 processor (Intel Corporation, Santa Clara, CA, USA) and 32 GB of RAM. Figure 4 presents a comparison of the point cloud data before and after preprocessing.

### 3.3. Verification Method

**Figure 5:**
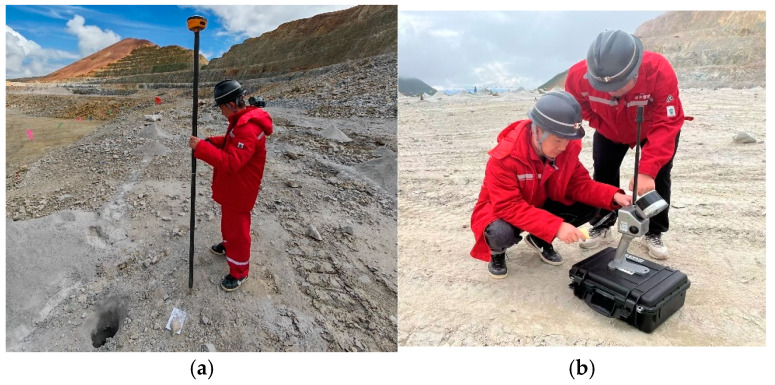
Verification Equipment. (**a**) Hi-Target Handheld RTK V200; (**b**) Jishang Navigation GS-102G 3D Laser Scanner.

To validate the accuracy of the proposed method, the minimum burden calculated from the point cloud model acquired by the DJI Matrice 300 RTK UAV (Manufactured by SZ DJI Technology Co., Ltd., Shenzhen, Guangdong, China) was compared against the ground truth value. This reference value was derived using a method combining handheld RTK (Manufactured by SZ DJI Technology Co., Ltd., Shenzhen, Guangdong, China) for blast hole positioning and a 3D laser scanner (manufactured by Jishang Navigation Technology Co., Ltd., Beijing, China) for capturing slope surface point cloud data. A quantitative error analysis was performed to verify the precision of the extracted minimum burden. All datasets involved in this study were transformed into a unified coordinate system prior to analysis. The UAV-derived point cloud acquired by the DJI M300 RTK system was directly georeferenced using onboard GNSS/RTK positioning, providing coordinates in a global reference frame. The blast-hole collar coordinates measured using the handheld RTK device share the same coordinate reference system, ensuring spatial consistency with the UAV point cloud. For the ground truth slope surface, the point cloud obtained from the 3D laser scanner was registered to the same coordinate system using RTK-measured reference points placed within the scanning area. These control points were used to align the laser scanning data with the UAV-derived point cloud, thereby ensuring consistency across all datasets. The Hi-Target Handheld RTK V200 (manufactured by Hi-Target Surveying Instrument Co., Ltd., Guangzhou, Guangdong, China) and the Jishang Navigation GS-102G 3D laser scanner (manufactured by Jishang Navigation Technology Co., Ltd., Beijing, China) used for ground truth data collection are shown in Figure 5.

The specific workflow of the aforementioned verification method is as follows: Firstly, the front-row blast holes within the blasting area are selected as validation samples. The slope surface area corresponding to each blast hole is scanned using the Jishang Navigation GS-102G 3D laser scanner (manufactured by Jishang Navigation Technology Co., Ltd., Beijing, China) (with a ranging accuracy of ±5 mm) to acquire a high-density, true point cloud of the slope surface. Concurrently, the top-center coordinates of each blast hole are measured with the Hi-Target Handheld RTK V200 device (manufactured by Hi-Target Surveying Instrument Co., Ltd., Guangzhou, Guangdong, China) (featuring a planar accuracy of ±8 mm and an elevation accuracy of ±15 mm). Subsequently, the geometric center coordinate $\left(x_{O}^{\mathrm{true}},y_{O}^{\mathrm{true}},z_{O}^{\mathrm{true}}\right)$ of the explosive column is calculated by combining these measurements with the explosive segment parameters from the engineering log. Based on the measured explosive center coordinates and the true slope surface point cloud $\left\{S_{k}^{\mathrm{true}}(x_{k}^{\mathrm{true}},y_{k}^{\mathrm{true}},z_{k}^{\mathrm{true}})\right\}$, the true minimum burden $W_{\mathrm{true}}$ is calculated using the Euclidean distance formula, where

(21) $$W_{\mathrm{true}}=\min\left\{\sqrt{{\left(x_{O}^{\mathrm{true}}-x_{k}^{\mathrm{true}}\right)}^{2}+{\left(y_{O}^{\mathrm{true}}-y_{k}^{\mathrm{true}}\right)}^{2}+{\left(z_{O}^{\mathrm{true}}-z_{k}^{\mathrm{true}}\right)}^{2}}\right\}$$

Subsequently, the UAV point cloud from the verification area is processed using the proposed method to obtain the calculated minimum burden $W_{\mathrm{calc}}$. The deviation is quantified using two metrics: absolute error and relative error:

(22) $$\Delta W_{\mathrm{abs}}=\left|W_{\mathrm{calc}}-W_{\mathrm{true}}\right|$$

(23) $$\Delta W_{\mathrm{rel}}=\frac{\Delta W_{\mathrm{abs}}}{W_{\mathrm{true}}}\times 100\%$$

Finally, the computational accuracy and reliability of the proposed method in engineering scenarios are evaluated by comparing the metrics across all validated blast holes.

### 3.4. Comparative Schemes and Evaluation Metrics

To evaluate the influence of each processing stage in the proposed method on the accuracy of slope surface extraction and minimum burden calculation, the region-growing algorithm and the DBSCAN algorithm are not treated as completely independent parallel approaches. Instead, staged comparative schemes are constructed according to the technical workflow proposed in this study. The detailed comparison schemes are summarized in Table 1.

In terms of evaluation metrics, the absolute error and relative error between the measured values and the calculated values of the minimum burden are adopted as quantitative indicators for assessing extraction accuracy. The corresponding calculation formulas are given in Equations (22) and (23). The absolute error reflects the direct deviation between the calculated and measured values, while the relative error is used to evaluate the error level under different blast-hole scales.

On the basis of the above indicators, to further reflect the dispersion of errors among different blast holes and the engineering applicability of the method, the standard deviation of absolute errors, the standard deviation of relative errors, the maximum absolute error, and the number of blast holes exceeding the tolerance threshold were additionally calculated. The standard deviations of absolute and relative errors were used to evaluate the fluctuation of errors among blast holes; smaller values indicate more stable calculation results. The maximum absolute error was used to reflect the deviation of the algorithm under the most unfavorable blast-hole condition. Considering the accuracy requirement of burden determination in medium-length hole blasting pattern design, 0.10 m was adopted as the engineering tolerance threshold. The number of blast holes with absolute errors greater than 0.10 m was counted to evaluate the reliability of the method in engineering applications.

## 4. Experimental Results

### 4.1. Regional Growth Process

**Figure 6:**
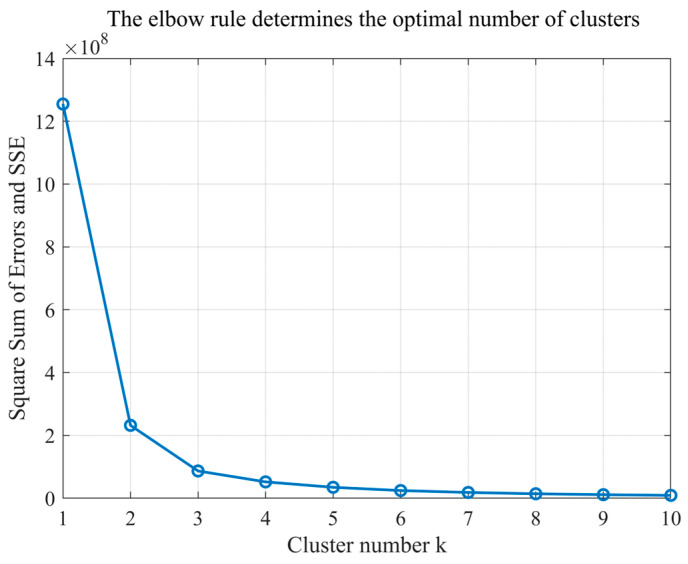
Elbow Method Curve.

**Figure 7:**
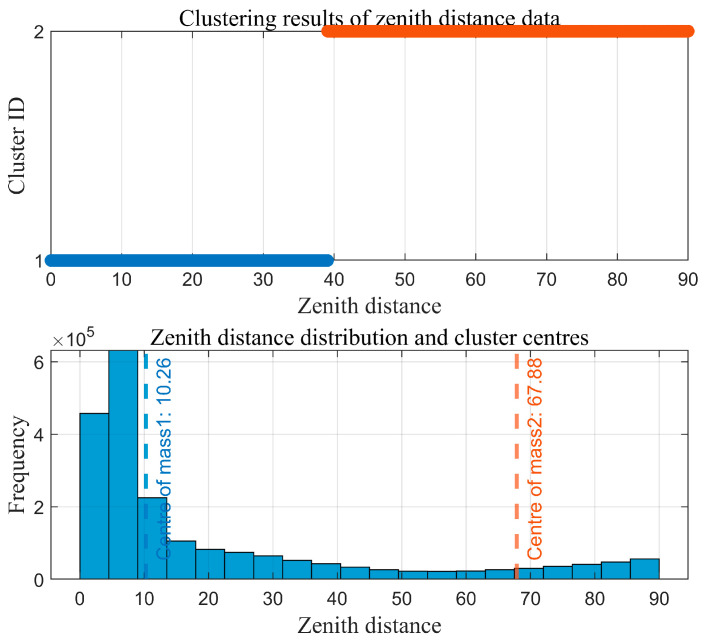
Clustering Results Distribution.

As the core of the region-growing algorithm, the quality of initial seed points directly determines the accuracy of subsequent slope surface extraction. It is essential to screen seed points based on geometric features that align with actual site conditions to ensure they genuinely reflect the spatial attributes of the slope. Considering the significant clustering characteristics of zenith angles in open-pit mine point clouds—where slope surfaces exhibit concentrated zenith angle distributions due to stable inclination, while non-slope areas (e.g., crest platforms, rock protrusions) show dispersed zenith angles—the optimal number of clusters is determined using the elbow method. The Within-Cluster Sum of Squares (WCSS) for different cluster numbers (k = 1~10) is calculated, and the inflection point in the WCSS curve is identified to determine the optimal k-value. This provides a basis for distinguishing between slope and non-slope point clouds. The elbow method curve and the distribution of clustering results involved in the initial seed extraction process are shown in Figure 6 and Figure 7, respectively.

As shown in Figure 6, when k increases from 1 to 2, the WCSS exhibits a marked decrease, indicating that clustering at this stage effectively separates point clouds with distinct zenith angle characteristics. However, when k > 2, the rate of decrease in WCSS plateaued significantly, suggesting that further increasing the number of clusters fails to enhance the clustering effectiveness and would instead introduce unnecessary computational overhead. Therefore, the optimal number of clusters is determined as 2.

Further analysis of the clustering results in Figure 7 reveals that Cluster 1, with a zenith angle range of $\left[0.00{}^{\circ},39.07{}^{\circ}\right]$ and a centroid at 10.26°, comprises a large number of data points characterized by relatively small zenith angles. Based on the topographic features of open-pit mines, this cluster can be identified as non-slope point clouds, such as crest platforms and flat areas. In contrast, Cluster 2, with a zenith angle range of $\left[39.07{}^{\circ},90.00{}^{\circ}\right]$ and a centroid at 67.88°, exhibits larger and more concentrated zenith angles. This pattern aligns with the typical zenith angle characteristics of inclined slope surfaces. Thus, the zenith angle range $\left[39.07{}^{\circ},90.00{}^{\circ}\right]$ corresponding to Cluster 2 is defined as the effective interval for slope surfaces, serving as the primary constraint for seed point screening.

**Figure 8:**
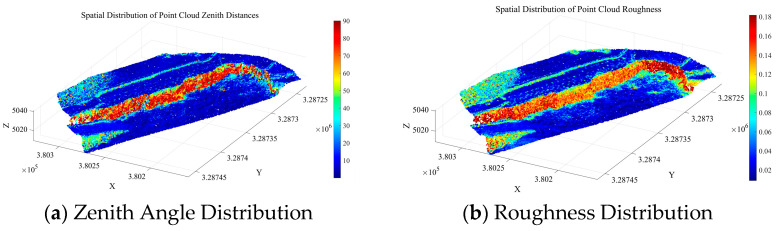
Distributions of Zenith Angle and Roughness in the Open-pit Mining Area.

**Figure 9:**
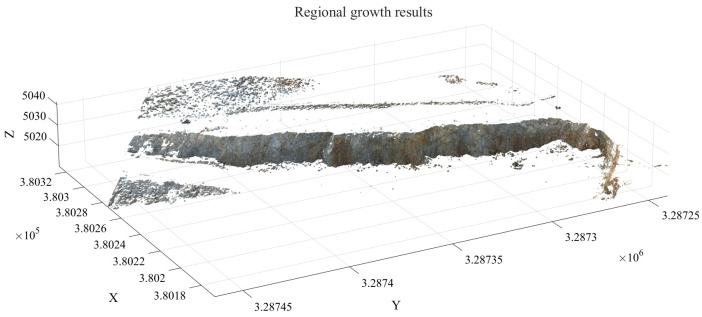
Region-Growing Results.

To further eliminate points corresponding to surface irregularities, such as rock protrusions and blasting remnants, within Cluster 2, a roughness index is introduced for secondary screening. The roughness is calculated using the K-nearest neighbors (KNN) method (with k= 30), which quantifies surface undulation by computing the standard deviation of elevation values within the neighborhood of each point. Based on the statistical characteristics of the roughness in Cluster 2, the screening threshold is set as the sum of the mean and standard deviation of the cluster’s roughness values. Points with roughness below this threshold are retained. The distribution of zenith angles and roughness in the open-pit mining area is shown in Figure 8. The initial seed growing results are presented in Figure 9.

After screening based on zenith angle and roughness criteria, a total of 43,941 initial seed points were obtained. Using these as a foundation, region growing was subsequently performed, yielding 366,993 slope surface point clouds. These results provide a foundational basis for subsequent slope surface extraction and minimum burden calculation.

### 4.2. Slope Surface Extraction and Optimization Results

**Figure 10:**
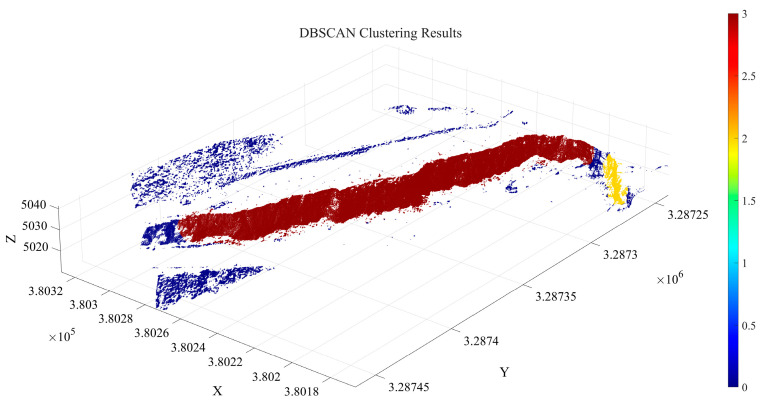
Distribution of Initial Slope Surface Clusters.

The primary objective of slope surface extraction and optimization is to remove pseudo slope-surface clusters, isolated noise points, and redundant boundary points from the point cloud clusters obtained through region growing. This process ensures the accuracy of the extracted free surface and provides a reliable spatial reference for subsequent minimum burden extraction. In this stage, the DBSCAN algorithm is employed for density optimization. Specifically, 500 sample points are randomly selected, and the average K-nearest neighbor distance is calculated. The sample mean distance is 0.21 m, with a standard deviation of 0.09 m. Accordingly, the neighborhood radius is set to ε = 0.3 m. To further evaluate the stability of the ε determination, the random sampling procedure was repeated multiple times using different sampling realizations. The results show that the estimated ε values fluctuate only slightly around 0.30 m, and the corresponding slope-surface extraction and minimum burden calculation results remain nearly unchanged. This indicates that the ε determination based on 500 randomly sampled points is stable and has a negligible influence on the final results. The minimum cluster size MinPts is adaptively determined based on the spatial density of the slope-surface point cloud. The total number of slope-surface points is Nc = 366,993, and the corresponding bounding box volume is V_bbox_. According to Equations (16)–(18), the resulting MinPts value in this experiment is 5. The distribution of the initial slope-surface clusters involved in this process is illustrated in Figure 10.

**Figure 11:**
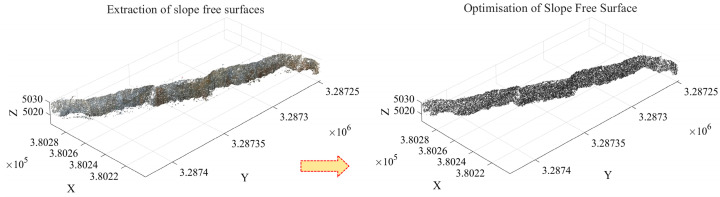
Slope Surface Optimization.

As shown in Figure 10, the proposed method identifies two valid clusters and 21,560 noise points, accounting for 5.87% of the total points. Among them, the largest cluster contains 302,180 points, representing 82.34% of the total points after clustering. However, the largest cluster still includes a small number of redundant points from the slope crest platform and debris points near the slope toe. To address this issue, the extent of the free surface is further constrained using the slope crest line and slope toe line. Specifically, the slope point cloud of the largest cluster is first projected onto the X–Y plane, and an image is constructed using elevation values as grayscale intensity. Subsequently, the Canny operator is applied for edge detection. After Gaussian filtering (*σ* = 1.2) and non-maximum suppression, the slope crest line and slope toe line are extracted. Finally, based on the extracted boundary lines, the coordinates of candidate slope-surface points are constrained to lie within the spatial region defined by the two boundaries, as illustrated in Figure 11.

As shown in Figure 11, the proposed method effectively removes anomalous point cloud data at the top and bottom of the slope surface. The optimized slope surface point cloud comprises a total of 295,420 points. This refined extraction and optimization of the slope surface lays a solid foundation for the subsequent calculation of the minimum burden.

### 4.3. Results Analysis of Different Comparative Schemes

After completing the slope surface extraction and optimization, the final 295,420 slope-surface points were used as the free-surface dataset. Based on the UAV point cloud model acquired by the DJI M300 RTK (Manufactured by SZ DJI Technology Co., Ltd., Shenzhen, Guangdong, China), together with parameters including blast-hole coordinates, hole radius, hole depth, and stemming length, a cylindrical three-dimensional blast-hole model was constructed. The geometric center of the explosive charge was determined as the average elevation of the upper and lower boundaries of the explosive section.

Subsequently, the Euclidean distance formula was used to calculate the shortest spatial distance between the charge center and each point on the free surface. This distance was taken as the calculated value of the minimum burden for the corresponding blast hole, denoted as *W*_*calc*_. The true value *W*_*true*_ was obtained using the actual slope-surface point cloud acquired through GS-102G 3D laser scanning (manufactured by Jishang Navigation Technology Co., Ltd., Beijing, China) combined with the blast-hole collar coordinates measured by a handheld RTKV200 device (manufactured by Hi-Target Surveying Instrument Co., Ltd., Guangzhou, Guangdong, China). Based on these datasets, the minimum burden of 22 blast holes located in the southern front row of the blasting area was calculated under the three processing schemes defined in Section 3.4, and the results were compared with the measured values for error analysis.

Among these schemes:

- Scheme A uses only the region-growing algorithm to extract the slope surface;
- Scheme B introduces DBSCAN-based density optimization on the basis of region growing;
- Scheme C represents the complete method proposed in this study.

**Figure 12:**
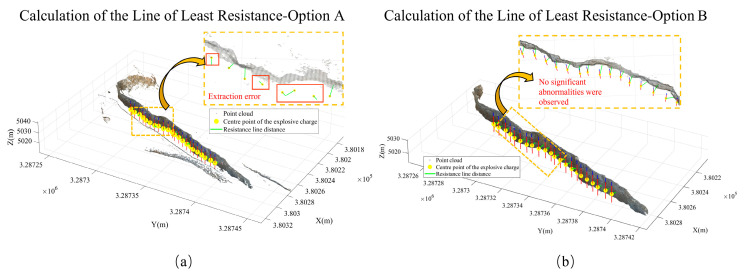
Illustration of minimum burden calculation results for Scheme A and B.

**Figure 13:**
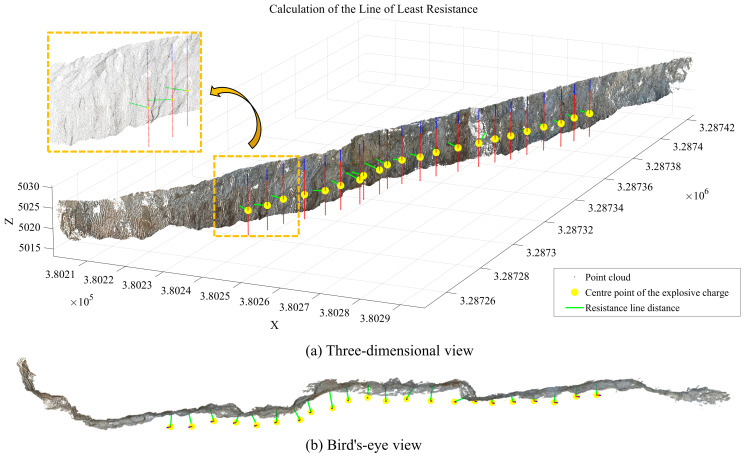
Minimum burden obtained using the proposed method (Scheme C).

Figure 12a,b illustrates the results of free-surface extraction and minimum burden calculation obtained using Scheme A and Scheme B, respectively, while Figure 13 presents the results obtained using the proposed method.

Through the calculation results of the three schemes, it can be observed that the region-growing algorithm can achieve the preliminary extraction of the slope surface. However, local noise points and pseudo slope-surface clusters still exist, which lead to deviations in the calculated minimum burden for some blast holes. After introducing DBSCAN-based density optimization, the isolated noise points are effectively removed, and the calculated minimum burden becomes closer to the measured values. On this basis, by further incorporating the slope crest and slope toe constraints, although the calculated values for some blast holes remain unchanged, the slope-surface boundary is more reasonably constrained. This effectively avoids the potential interference of crest platforms and debris accumulation near the slope toe on free-surface identification, thereby improving the stability of the proposed method in complex engineering scenarios. Furthermore, statistical analysis of the minimum burden obtained from Schemes A, B, and C and the corresponding measured values is presented in Table 2. The error statistics of different schemes at the Huatailong open-pit mine are shown in Table 3.

As shown in Table 2, the three schemes exhibit clear hierarchical differences in the extracted minimum burden results. In Scheme A, six blast hole-Nos. 6, 8, 13, 15, 18, and 22-show relatively large deviations, with absolute errors exceeding 1 m. This indicates that when the free surface is extracted solely using the region-growing algorithm, local abnormal points and pseudo slope-surface clusters may still interfere with the shortest-distance determination, resulting in distorted minimum burden calculations for some blast holes. After introducing DBSCAN-based density optimization in Scheme B, the errors associated with these abnormal blast holes are significantly corrected. The mean absolute error decreases from 0.55 m to 0.08 m, while the mean relative error decreases from 15.54% to 2.69%, demonstrating that density optimization plays a significant role in improving free-surface recognition quality and suppressing abnormal errors. The overall results of Scheme C are close to those of Scheme B, further indicating that after noise removal, the proposed method provides stable and consistent minimum burden calculations.

As shown in Table 3, Scheme A not only exhibits a relatively large mean error, but also has a standard deviation of absolute error of 0.83 m and a standard deviation of relative error of 22.75%. This indicates that, when only the region-growing algorithm is used, the errors vary considerably among different blast holes, and some blast holes are significantly affected by pseudo-slope surfaces and isolated noise points. The maximum absolute error of Scheme A reaches 2.57 m, and 9 blast holes exceed the 0.10 m threshold, indicating a relatively high computational risk in locally complex areas. After introducing DBSCAN, the standard deviation of absolute error in Scheme B decreases to 0.04 m, the maximum absolute error decreases to 0.21 m, and the number of blast holes exceeding 0.10 m is reduced to 4. This suggests that density optimization can effectively weaken the influence of abnormal points on the shortest-distance determination. Scheme C is generally close to Scheme B, with mean absolute errors of 0.077 m and 0.078 m, a mean relative error of approximately 2.68%, and a standard deviation of absolute error of 0.04 m, indicating that the results remain stable after introducing the crest–toe line constraints.

### 4.4. Applicability Analysis in Different Mining Areas

To further verify the applicability of the proposed method under different mining terrain conditions, the Qianshan Limestone Open-Pit Mine in Liaoyang and the Qidashan Iron Open-Pit Mine in Anshan were selected as additional validation sites. The true minimum burden values for both mines were obtained using the same approach described in Section 3.3, where blast-hole coordinates were measured in the field and combined with the actual slope-surface point cloud data. The two mining areas differ significantly in terms of bench structure, slope morphology, and blast-hole layout scale. In the Qianshan limestone open-pit mine, the slope surface is relatively gentle and the bench morphology is relatively regular, with a single bench height of approximately 16 m, and 17 blast holes arranged within the validation area. In contrast, the Qidashan iron open-pit mine exhibits a more complex slope structure, where interrupted benches and the connection of large and small benches are common. The single bench height is approximately 30 m, and 19 blast holes are arranged within the validation area. These differences allow the two mines to represent two typical engineering scenarios: gently inclined, regular slopes and high-bench, complex composite slopes. Consequently, they provide a suitable basis for evaluating the adaptability of the proposed method under different open-pit mining terrain conditions. The detailed results are presented in Table 4.

As shown in the results presented in Table 4, under two different mining conditions—the Qianshan Limestone Open-Pit Mine in Liaoyang and the Qidashan Iron Open-Pit Mine in Anshan—the proposed method can stably identify the main slope structures. Specifically, in the Qianshan limestone open-pit mine, the extracted slope surfaces are generally continuous, and the slope boundaries obtained after region growing and density optimization are relatively clear. In contrast, although the Qidashan iron open-pit mine exhibits a more complex bench configuration, the combination of region growing and DBSCAN-based density optimization can still effectively identify the main slope surfaces and form a complete slope-surface spatial extent. Overall, the results demonstrate that the proposed method exhibits strong slope-surface recognition capability and engineering applicability under different mining conditions. On this basis, to further evaluate the influence of different processing schemes on the minimum burden calculation, the minimum burden values were calculated using Schemes A, B, and C, respectively. The measured values were used as reference values, and the error statistics are presented in Table 5.

Under different mining conditions, the noise proportions identified by DBSCAN show a trend consistent with the complexity of the slope structure. In the slope surface point cloud of the Huatailong open-pit mine, the identified noise points account for 5.87%, mainly consisting of scattered abnormal points and local rock protrusions, which cause certain interference with the continuity of the free surface. The slope of the Liaoyang Qianshan limestone open-pit mine is generally gentle, with a regular bench morphology and good spatial continuity of the point cloud. The noise is mainly concentrated in scattered noise points, locally scattered rocks, and platform edges, resulting in a relatively low overall noise proportion of approximately 4.32%. In contrast, the slope structure of the Anshan Qidashan open-pit iron mine varies considerably, with complex bench connections and frequent superposition of local faults and multi-level benches. As a result, the number of unstructured points in the point cloud increases. In addition to scattered noise points, interference from platform edges and rock accumulation areas is more prominent, and the noise proportion increases to 7.46%. The comparison shows that the more complex the slope morphology and the more intense the local structural variation, the higher the proportion of noise points and the more obvious the influence on free-surface extraction. Combined with the error statistics, in the Qidashan mining area with stronger noise interference, the optimized method can still control the mean absolute error at 0.08 m, which is significantly lower than that obtained using only region growing. This indicates that the method can effectively reduce the influence of scattered noise points, rocks, and platform-edge points on minimum burden calculation, thereby ensuring the stability of the results under complex slope conditions.

As shown in Table 5, the three schemes exhibit clear differences in accuracy for minimum burden extraction across the two mining areas. When Scheme A, which relies solely on the region-growing algorithm, is applied, the results are influenced by noise clusters, isolated noise points, and pseudo slope-surface regions. Consequently, approximately one-third of the blast holes show relatively large deviations. For the remaining blast holes, where the shortest-distance points lie on relatively stable main slope surfaces, the calculated results of the three schemes are generally consistent. In the Qianshan Limestone Open-Pit Mine in Liaoyang, the mean absolute error of Scheme A is approximately 0.21 m, whereas in the Qidashan Iron Open-Pit Mine in Anshan it increases to approximately 0.30 m, indicating that a single algorithm is more likely to produce larger errors under complex slope conditions. After introducing DBSCAN-based density optimization, Scheme B effectively removes isolated noise points and corrects abnormal blast-hole results. As a result, the mean absolute errors in the two mining areas decrease to approximately 0.06 m and 0.10 m, respectively, demonstrating a significant improvement in overall accuracy. By further incorporating the slope crest and slope toe constraints, Scheme C produces results that are largely consistent with those of Scheme B. However, for blast holes located in areas with complex boundary conditions, additional corrections can still be observed. In this case, the mean absolute error in the Qianshan limestone open-pit mine remains approximately 0.06 m, while that in the Qidashan iron open-pit mine is further reduced to about 0.08 m. These experimental results indicate that the slope crest–toe constraint has only a minor influence on regular slope geometries. However, under complex combined-bench conditions, it can further improve the accuracy of free-surface boundary identification, thereby enhancing the stability and reliability of minimum burden extraction.

As shown in Table 6, the two validation mines, Liaoyang Qianshan and Anshan Qidashan, show the same trend. At Liaoyang Qianshan, where the slope morphology is relatively regular, Scheme C achieves a mean absolute error of 0.06 m and a maximum absolute error of 0.09 m, with no blast holes exceeding 0.10 m. At Anshan Qidashan, where the slope structure is more complex, the maximum absolute error of Scheme A reaches 0.75 m, and 7 blast holes exceed 0.10 m, indicating that the standalone region-growing algorithm is insufficiently stable in combined bench areas and locally complex boundary regions. After introducing DBSCAN in Scheme B, the mean error is markedly reduced, although 7 blast holes still exceed 0.10 m. After adding crest–toe boundary constraints in Scheme C, the mean absolute error decreases to 0.08 m, the standard deviation of absolute error decreases to 0.01 m, and the number of blast holes exceeding 0.10 m decreases to 2. These results indicate that boundary constraints have only a limited influence on regular slopes, but can further reduce boundary errors and improve the stability of minimum burden extraction under complex combined bench conditions.

To examine the stability of the elbow method in different mining areas, k-means clustering analysis was performed on the zenith-angle distributions of the Huatailong, Liaoyang Qianshan, and Anshan Qidashan mining areas. The value of k was set from 1 to 5 for all three sites, and the optimal number of clusters was determined according to the variation in the WCSS curve. The experimental results show that all three mining areas exhibit a clear decrease followed by a gradual flattening around k = 2, indicating that the zenith-angle distribution of the point cloud can mainly be divided into non-slope regions with low zenith angles and slope regions with high zenith angles. For the Qidashan mining area, where the local slope structure is relatively complex, setting k = 3 can further subdivide some transition areas. However, the additional cluster mainly corresponds to local variations near the slope crest and toe, and has little influence on the identification of the main slope-surface range. To maintain consistent processing standards across different mining areas and avoid slope-surface fragmentation caused by over-segmentation, the dominant slope cluster was uniformly selected as the seed-point range for subsequent processing. When multiple candidate slope clusters appeared in a local mining area, the cluster with a larger zenith angle, better spatial continuity, and consistency with the position of the free surface in front of the blast holes was selected as the slope-surface cluster. The experimental results show that this strategy does not reduce the accuracy of subsequent minimum burden calculation. The mean absolute errors of Scheme C in the Liaoyang Qianshan and Anshan Qidashan mining areas are 0.06 m and 0.08 m, respectively, indicating that the main slope-surface range determined by the elbow method has good stability.

## 5. Discussion

### 5.1. Influence Analysis of Preprocessing Errors on Results

Point cloud preprocessing alters the local geometric morphology of the slope. The associated errors mainly originate from Octree downsampling, progressive morphological filtering, and the measurement accuracy of validation equipment. To analyze how these errors affect the calculation of the minimum burden, preprocessing errors are categorized into planar position errors, elevation errors, boundary extraction errors, and reference measurement errors. Since the minimum burden is defined as the shortest Euclidean distance from the explosive charge center to the slope free surface, these errors ultimately manifest as variations in the coordinates of either the free-surface points or the charge center, thereby further influencing the calculated minimum burden.

In Octree downsampling, the centroid of each voxel is used to represent all original points within the voxel. Let the voxel edge length be *l*. The theoretical maximum positional deviation of a point along the *x*, *y*, and *z* directions can be expressed as:

(24) $$\Delta x_{\mathrm{oct}}\leq \frac{l}{2},\;\Delta y_{\mathrm{oct}}\leq \frac{l}{2},\;\Delta z_{\mathrm{oct}}\leq \frac{l}{2}$$

where Δ*x*_oct_, Δ*y*_oct_, Δ*z*_oct_ denote the maximum coordinate deviations introduced by Octree downsampling in the *x*, *y*, and *z* directions, respectively, and *l* is the voxel edge length. In this study, *l* = 0.08 m, yielding a theoretical maximum deviation of 0.04 m in each direction. This error mainly affects local surface details and has a relatively limited influence on the overall slope geometry.

The progressive morphological filtering method identifies non-geological objects based on elevation differences. During the filtering process, if the structuring element becomes excessively large, partial weakening may occur in regions such as the slope crest, slope toe, and areas with abrupt elevation changes. Let the elevation of a point before filtering be *z*_*i*_, and after filtering be $z_{i}^{\prime }$; the elevation error introduced by filtering can be expressed as:

(25) $$\Delta z_{\mathrm{pmf},i}=z_{i}-z_{i}^{\prime }$$

where Δ*z*_*pmf*,*i*_ represents the elevation variation in the *i*-th point after progressive morphological filtering; *z*_*i*_ and $z_{i}^{\prime }$ denote the elevations before and after filtering, respectively. To characterize the overall impact on the slope surface, the mean elevation error and the maximum elevation error are defined as:

(26) $$\Delta {\bar{z}}_{\mathrm{pmf}}=\frac{1}{N_{p}}\sum_{i=1}^{N_{p}}|\Delta z_{\mathrm{pmf},i}|$$

(27) $$\Delta z_{\mathrm{pmf},\max}=\max\left(\left|\Delta z_{\mathrm{pmf},i}\right|\right)$$

where $\Delta {\bar{z}}_{\mathrm{pmf}}$ denotes the mean elevation error introduced by morphological filtering, Δ_*zpmf*,max_ is the maximum elevation error, and *N*_*p*_ is the number of slope surface points involved in the statistics. These metrics reflect the degree to which preprocessing weakens the elevation morphology of the slope.

Boundary errors mainly arise from differences in the removal and preservation of points near the slope crest and toe. Let the boundary point set extracted before preprocessing be *L*_*raw*_, and after preprocessing be *L*_*proc*_. The average boundary displacement can be expressed as:

(28) $$\Delta {\bar{d}}_{b}=\frac{1}{N_{b}}\sum_{j=1}^{N_{b}}\underset{q_{j}\in L_{\mathrm{proc}}}{\min}\|p_{j}-q_{j}\|$$

where $\Delta {\bar{d}}_{b}$ represents the average displacement of the boundary before and after preprocessing; *p*_*j*_ is the *j*-th boundary point in *L*_*raw*_; *q*_*j*_ is the nearest point in *L*_*proc*_ to *p*_*j*_; and *N*_*b*_ is the number of boundary points.

In the minimum burden calculation, the geometric center of the explosive charge is denoted as *O*(*x*_0_, *y*_0_, *z*_0_), and the slope free-surface points are denoted as *Pk*(*x*_*k*_, *y*_*k*_, *z*_*k*_). When preprocessing alters the coordinates of slope points, the variation in the minimum burden for the same blast hole can be expressed as:

(29) $$\Delta W_{\mathrm{pre}}=\left|\underset{P_{k}^{\prime }\in S_{\mathrm{proc}}}{\min}∥O-P_{k}^{\prime }∥-\underset{P_{k}\in S_{\mathrm{raw}}}{\min}\|O-P_{k}\|\right|$$

where Δ*W*_*pre*_ denotes the change in the minimum burden caused by preprocessing errors; *S*_*raw*_ is the free-surface point set extracted from the raw point cloud; *S*_*proc*_ is the free-surface point set after Octree downsampling and progressive morphological filtering; *P*_*k*_ and $P_{k}^{\prime }$ represent free-surface points before and after preprocessing, respectively; and *O* is the geometric center of the explosive charge. It is assumed that blast-hole design parameters are not affected by preprocessing. Therefore, variations in *O* are mainly determined by RTK measurement errors, while preprocessing errors are primarily propagated through the free-surface point set *S*_*proc*_.

Considering the accuracy of validation equipment, the planar accuracy of the handheld RTK is ±0.008 m, the elevation accuracy is ±0.015 m, and the ranging accuracy of the 3D laser scanner is ±0.005 m. The uncertainty of the reference value can be approximated as:

(30) $$u_{\mathrm{true}}=\sqrt{u_{xy}^{2}+u_{z}^{2}+u_{\mathrm{scan}}^{2}}$$

where *u*_*true*_ represents the uncertainty of the true minimum burden reference value; *u*_*xy*_ denotes RTK planar positioning error; *u*_*z*_ represents RTK elevation error; and *u*_*scan*_ denotes the ranging error of the 3D laser scanner. Substituting the equipment specifications yields:

(31) $$u_{\mathrm{true}}=\sqrt{0.008^{2}+0.015^{2}+0.005^{2}}=0.0177\;m$$

Therefore, the experimental reference values exhibit an inherent measurement uncertainty of approximately 0.018 m. This value is significantly smaller than the mean absolute error of 0.077 m obtained in the Huatailong open-pit mine experiments, indicating that the primary source of computational error originates from free-surface identification and point cloud preprocessing rather than measurement equipment.

To further quantify the impact of preprocessing errors on the final minimum burden, 22 blast holes on the 5015 platform of the Huatailong open-pit mine were selected. Calculations were performed using raw point clouds, Octree-downsampled point clouds, and fully preprocessed point clouds, respectively, and the variations in minimum burden before and after preprocessing were statistically analyzed. The results are summarized in Table 7.

As shown in Table 7, the mean value of the total propagated error is 0.039 m, which is smaller than the mean absolute error of 0.077 m observed in the Huatailong open-pit mine experiments. This indicates that although preprocessing introduces local geometric deviations, the magnitude of error propagation remains within a controllable range. Combined with the comparison of point clouds before and after preprocessing (Figure 4), it can be observed that preprocessing mainly removes non-geological objects such as pickup trucks, personnel, and construction equipment, while preserving the main geometric structure of the slope. Therefore, it does not significantly alter the free-surface geometry on which the minimum burden calculation depends.

### 5.2. Comparative Analysis with the Improved DBSCAN Method

To further investigate the performance of the proposed method, the improved DBSCAN method proposed by Liu et al. [16] is selected as an external comparative approach. This method determines clustering thresholds through point cloud completion and DDM-based density distribution, and has been applied to structural surface extraction in hard rock mining, demonstrating strong clustering and noise identification capabilities. In this study, the method is applied to slope extraction on the 5015 platform of the Huatailong open-pit mine. The minimum burden is calculated under identical conditions of blasthole coordinates, blasthole radius, blasthole depth, and stemming length.

**Figure 14:**
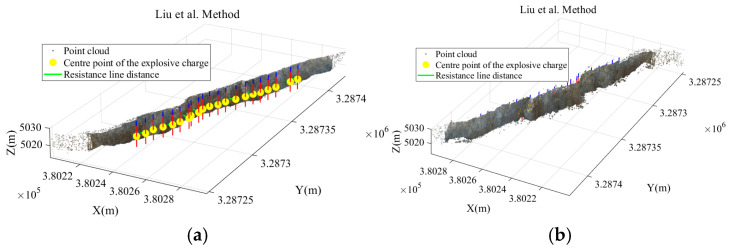
Minimum burden Extraction Using the Improved Method of Liu et al. (**a**) Visualisation of minimum resistance line extraction; (**b**) Detailed view of slope extraction.

The key parameter settings are as follows: the global neighbourhood radius for point cloud completion is set to 0.30 m, the DDM grid size is 0.25 m, the initial DBSCAN neighbourhood radius is 0.28 m, and MinPts is 5. The parameters of the proposed method remain consistent with those in Section 4.2, i.e., the DBSCAN neighbourhood radius is 0.30 m and MinPts is 5. Both methods use the measured minimum burden as the reference value, and are evaluated using mean absolute error, mean relative error, maximum absolute error, and the number of blastholes with errors exceeding 0.10 m. The experimental results are shown in Table 8, and the visualisation is presented in Figure 14.

As shown in Table 8, the mean absolute error of the proposed method is 0.077 m, and the mean relative error is 2.68%, both of which are lower than those of the improved DBSCAN method by Liu et al. Although the improved DBSCAN method can effectively remove some low-density noise points and has a certain capability in identifying the main slope regions, it primarily relies on point cloud density distribution for clustering, and does not sufficiently consider zenith angle, roughness, and slope crest–toe boundary constraints. In open-pit bench slopes, the slope platform, accumulation at the slope foot, and local rock protrusions are often spatially connected to the actual slope surface. Relying solely on density differences may retain some platform or accumulation points, and may also weaken the integrity of slope boundaries in regions with significant density variation. The proposed method first uses zenith angle and roughness to select slope seed points, and then maintains the continuity of the slope surface through region growing. Subsequently, DBSCAN is applied to remove discrete noise, and slope crest and toe constraints are introduced to limit the range of the free surface. Therefore, the method can simultaneously ensure slope integrity and effective noise removal. The comparison results indicate that, compared with the method using improved DBSCAN alone, the proposed method achieves higher accuracy and stability in minimum burden extraction under complex open-pit slope conditions, and exhibits stronger engineering applicability.

### 5.3. Parameter Sensitivity Analysis

**Figure 15:**
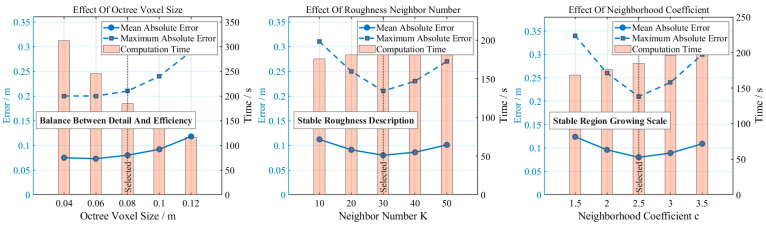
Visualisation of the Influence of Key Parameters.

To further analyse the influence of key parameters on the accuracy of minimum burden extraction, the first 22 blastholes on the 5015 platform of the Huatailong open-pit mine are selected as the study objects. The octree minimum voxel size, the number of neighbouring points K for roughness calculation, and the adaptive neighbourhood radius coefficient c are varied, while keeping other processing steps unchanged. The mean absolute error, maximum absolute error, and computation time are calculated under each condition. The octree voxel size mainly affects the preservation of point cloud details and the data volume; the value of K mainly influences the representation of local surface roughness; and the coefficient c mainly affects the scale of the normal vector neighbourhood and the continuity of region growing. The parameter sensitivity results are shown in Table 9, and the variation trends are illustrated in Figure 15.

As shown in Table 9 and Figure 15, variations in the three types of parameters all affect the extraction results, but in different ways. When the octree voxel size increases from 0.04 m to 0.12 m, the computation time continuously decreases, while the mean absolute error gradually increases after 0.08 m, indicating that excessively large voxels weaken the representation of slope crest, slope toe, and local concave–convex features. When the roughness neighbourhood size K is small, the results are easily affected by individual outliers; when K is large, the real surface undulations tend to be smoothed. The mean absolute error is minimised and the maximum absolute error is smallest when K = 30. When the coefficient c is small, the neighbourhood scale is insufficient and region growing may exhibit local fragmentation; when c is large, the neighbourhood range expands and non-target points near slope boundaries are more likely to be included. Considering accuracy, maximum error, and computation time, the optimal parameter combination is selected as voxel size 0.08 m, K = 30, and c = 2.5. Under this configuration, the mean absolute error is 0.077 m and the maximum absolute error is 0.21 m, which ensures computational efficiency while preserving the main geometric features of the slope surface.

Furthermore, it should be noted that although the three parameters (octree voxel size, neighbourhood size K, and coefficient c) influence the extraction results to varying degrees, the proposed method exhibits relatively stable performance within a reasonable parameter range. This is mainly attributed to the adaptive parameter determination strategy embedded in the algorithm, where the neighbourhood scale and clustering thresholds are derived from point cloud density characteristics rather than fixed empirical values. Specifically, when the voxel size varies within 0.06–0.10 m, K ranges from 20 to 40, and c ranges from 2.0 to 3.0, the mean absolute error fluctuates only slightly, indicating that the method is not highly sensitive to parameter perturbations. This robustness reduces the dependence on manual parameter tuning and enhances the applicability of the method in different open-pit mining scenarios. Therefore, the selected parameter combination (voxel size = 0.08 m, K = 30, c = 2.5) represents a balanced choice rather than a strictly optimal solution, ensuring both computational efficiency and extraction accuracy in practical engineering applications.

### 5.4. Limitations and Future Work

This study proposes an intelligent extraction method for the minimum burden in medium-deep hole blasting, based on the integration of region growing and an optimised DBSCAN algorithm. The method comprises three principal stages, namely feature-based filtering, region growing, and density refinement. It has been validated using data from the 5015 bench of the Huatailong open-pit mine in Tibet and further evaluated through comparative experiments in the Qianshan limestone quarry in Liaoyang and the Qidashan iron mine in Anshan. The results indicate that the proposed approach is capable of consistently identifying the principal slope structures under varying mining conditions, with the extracted minimum burden values demonstrating good agreement across different sites. In areas where slope morphology is relatively regular, the extracted surfaces are continuous and well-defined; in more complex bench environments, although local variations in slope geometry are evident, the combined application of region growing and DBSCAN effectively suppresses the influence of noise and pseudo-slope surfaces, thereby maintaining stable computational accuracy.

From a methodological perspective, region growing utilises point cloud normal vectors and spatial continuity to delineate slope surfaces, which is consistent with the approach reported by Liu et al. [16]. Building upon this, the incorporation of DBSCAN enables the removal of density anomalies, effectively eliminating local noise and pseudo-surfaces. This is in line with previous findings demonstrating the robustness of DBSCAN in complex point cloud environments [23]. Furthermore, the adaptive adjustment of parameters according to point cloud density mitigates the dependence on empirical settings. Comparative results show that when region growing is applied in isolation, certain blastholes exhibit significant errors due to inaccuracies in free-surface identification; the introduction of density refinement markedly reduces such anomalies. The subsequent incorporation of crest–toe constraints leads to only marginal changes in overall results, but improves stability in areas with complex boundaries, indicating that boundary constraints play a supplementary yet important role under such conditions.

Nevertheless, several limitations remain. It should also be clarified that the minimum burden extracted in this study represents a geometric minimum burden, defined as the shortest Euclidean distance from the geometric centre of the explosive charge to the reconstructed free surface. This definition is consistent with the point-cloud-based objective of the present method. However, in fractured bench slopes, the mechanically effective burden may be influenced not only by the geometric distance to the free surface, but also by discontinuity orientation, pre-existing damage, local rock-mass anisotropy, and the spatial distribution of weakness planes. Therefore, the value obtained by the proposed method should be interpreted as a geometric estimate and a practical proxy for the mechanically effective burden, rather than a direct representation of the actual path of least resistance during blasting. Further integration of discontinuity mapping, rock-mass structure characterization, and blasting-induced damage modelling would be required to quantify the mechanically effective burden more rigorously. The method assumes the presence of a clearly defined dominant slope surface; in cases where the rock mass is highly fragmented or intersected by multiple structural planes, discontinuities or misclassification of slope surfaces may occur. The blasthole is modelled as an idealised cylinder, without accounting for deviations such as hole inclination, diameter variation, or charge misalignment, which may introduce errors in the estimated charge centre. The data acquisition relies primarily on unmanned aerial vehicle (UAV) photogrammetry; under conditions of weak texture, occlusion, or blasting-induced dust, the resulting point clouds may suffer from data gaps or uneven density, thereby affecting the accuracy of free-surface identification. In addition, the number of validation sites remains limited, and the applicability of the method under more complex geological conditions requires further investigation. Future work may incorporate the integration of UAV imagery with LiDAR data to enhance data completeness in challenging environments, following the multi-source data fusion strategy proposed by Pu et al. [29]. Deep learning techniques may also be employed for adaptive parameter optimisation, as suggested by Yang et al. [30]. Furthermore, the introduction of point cloud quality assessment and post-blast fragmentation analysis, as discussed by Varbla et al. [31], could enable the development of a feedback-driven optimisation framework, thereby further improving both the accuracy and engineering applicability of minimum burden extraction.

In addition, from the standpoint of blasting mechanics, the minimum burden is not merely a geometric parameter, but a key factor influencing the direction of explosive energy release, the propagation of stress waves, and the resulting fragmentation performance. Previous studies [32] have shown that, in medium-deep hole blasting, the distribution of the blasting-induced stress field and the extent of the failure zone are jointly governed by the blasthole charge configuration and the structural conditions of the bench rock mass. Under conditions of developed fracturing or high benches, both stress propagation and failure-zone expansion exhibit pronounced heterogeneity, and the extent of the failure zone is closely coupled with the minimum burden. Consequently, accurate determination of the minimum burden is essential for controlling the effective range of blasting and optimising energy utilisation. For example, investigations into fractured high benches [33] indicate that the stress field generated by blasthole charges directly determines the morphology and extent of the failure zone, whose boundary is strongly constrained by the position of the free surface. In this context, the minimum burden, as the principal parameter governing the direction of energy release, directly affects the prediction of the failure zone and the control of blasting safety. Moreover, in practical blasting operations in typical limestone quarries, the minimum burden, together with blast-pattern parameters, governs the fragmentation size distribution; an appropriately designed burden contributes to improved fragmentation uniformity and reduced secondary breakage costs.

Based on the above considerations, the proposed intelligent extraction method for minimum burden not only achieves improved geometric accuracy in point cloud processing, but also provides a more reliable basis for blast design in engineering practice. By enhancing the accuracy of minimum burden estimation, the method has the potential to support the optimisation of blast-pattern parameters, improve fragmentation performance, and promote more efficient utilisation of explosive energy, thereby strengthening its practical value in open-pit blasting operations.

## 6. Conclusions

To address the difficulty of directly measuring the minimum burden in medium-length hole bench blasting in open-pit mines and the limited accuracy of single-algorithm approaches, this study proposes an automated extraction method based on UAV-derived 3D point cloud data, integrating region growing and DBSCAN optimization. The method combines zenith angle clustering, roughness filtering, region growing, DBSCAN-based density refinement, and crest–toe boundary constraints to extract slope free surfaces, followed by the computation of the shortest Euclidean distance between the explosive charge center and the free surface. The main conclusions are as follows:

(1) The proposed method can effectively identify slope free surfaces in open-pit mines. Region growing preserves the global geometric continuity of the slope, while DBSCAN removes discrete noise such as rock protrusions and blasting residues. The crest and toe constraints further refine the boundary of the free surface, reducing interference from platform areas and debris accumulation.
(2) An adaptive parameter determination strategy applicable to varying point cloud densities is established. The neighborhood scale for normal estimation, the DBSCAN neighborhood radius, and the minimum cluster size are all automatically derived from spatial distribution characteristics, thereby reducing the subjectivity associated with manual parameter selection. Validation results at the Huatailong open-pit mine show a mean absolute error of 0.077 m and a mean relative error of 2.68%, meeting engineering accuracy requirements.
(3) Comparative analysis of three processing schemes demonstrates that the standalone region-growing algorithm is sensitive to pseudo-slope surfaces and noise, leading to large errors in some blast holes. The introduction of DBSCAN significantly reduces abnormal errors, while the addition of crest–toe constraints further improves stability under complex bench conditions. Validation results from the Qianshan and Qidashan open-pit mines further confirm the robustness and engineering applicability of the proposed method, providing reliable support for blast-hole pattern design and digital blasting in open-pit mining.

## Figures and Tables

**Table 1:** Description of comparative schemes.

Scheme	Processing Method	Description
A	Region Growing	Only the region-growing algorithm is used to extract the initial slope-surface point cloud and calculate the minimum burden.
B	Region Growing + DBSCAN	Based on the region-growing results, DBSCAN density optimization is introduced to remove pseudo slope-surface clusters and isolated noise points, extract the largest continuous slope surface, and calculate the minimum burden.
C	Proposed Method	On the basis of Scheme B, additional constraints based on the crest and toe of the slope are incorporated to further refine the slope-surface boundary, followed by minimum burden calculation.

**Table 2:** Statistical comparison of minimum burden extraction results.

Blast Hole No.	True Value/m	Calculated Value of Proposed/m	Scheme A		Scheme B		Scheme C	
			Absolute Error/m	Relative Error/m	Absolute Error/m	Relative Error/m	Absolute Error/m	Relative Error/m
1	4.13	4.28	0.15	3.63%	0.15	3.63%	0.15	3.63%
2	1.71	1.75	0.04	2.57%	0.04	2.57%	0.04	2.57%
3	3.33	3.24	0.09	2.82%	0.09	2.82%	0.09	2.82%
4	4.37	4.41	0.04	0.94%	0.04	0.94%	0.04	0.94%
5	2.68	2.74	0.06	2.16%	0.06	2.16%	0.06	2.16%
6	3.57	3.64	1.70	47.62%	0.08	2.19%	0.07	1.96%
7	3.83	3.62	0.21	5.54%	0.21	5.54%	0.21	5.54%
8	3.57	3.52	1.30	36.41%	0.05	1.32%	0.05	1.32%
9	3.64	3.59	0.05	1.48%	0.05	1.48%	0.05	1.48%
10	4.06	3.97	0.09	2.19%	0.09	2.19%	0.09	2.19%
11	3.92	3.87	0.05	1.33%	0.05	1.33%	0.05	1.33%
12	3.56	3.62	0.06	1.71%	0.06	1.71%	0.06	1.71%
13	4.05	3.96	1.28	31.60%	0.09	2.22%	0.09	2.22%
14	2.2	2.13	0.07	3.36%	0.07	3.36%	0.07	3.36%
15	3.66	3.62	2.57	70.22%	0.04	1.23%	0.04	1.23%
16	3.52	3.47	0.05	1.53%	0.05	1.53%	0.05	1.53%
17	3.84	3.71	0.13	3.36%	0.13	3.36%	0.13	3.36%
18	5.02	4.88	1.88	37.45%	0.14	2.77%	0.14	2.77%
19	1.42	1.34	0.08	5.42%	0.08	5.42%	0.08	5.42%
20	0.57	0.60	0.03	5.26%	0.03	5.26%	0.03	5.26%
21	1.53	1.47	0.06	4.12%	0.06	4.12%	0.06	4.12%
22	3.01	2.95	2.14	71.10%	0.06	2.06%	0.06	2.06%
MAE	/	/	0.550	15.54%	0.078	2.69%	0.077	2.68%

**Table 3:** Error Statistics of Different Schemes at the Huatailong Open-pit Mine.

Scheme	Mean Absolute Error/m	Standard Deviation of Absolute Error/m	Maximum Absolute Error/m	Mean Relative Error/%	Standard Deviation of Relative Error/%	Number of Blast Holes Exceeding 0.10 m
Scheme A	0.55	0.83	2.57	15.54	22.75	9
Scheme B	0.08	0.04	0.21	2.69	1.40	4
Scheme C	0.07	0.04	0.21	2.68	1.41	4

**Table 4:** Illustration of minimum burden extraction in the Qianshan and Qidashan open-pit mining areas.

Mining Area	Preprocessing Results	Zenith Angle Distribution	Roughness Distribution	Slope Surface Extraction	Slope Surface Optimization	Minimum Burden Extraction
Qianshan, Liaoyang	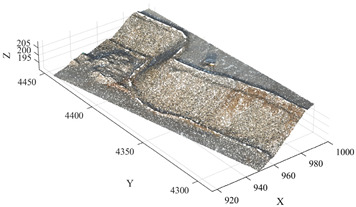	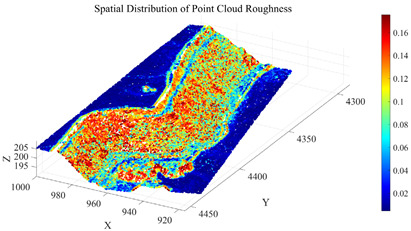	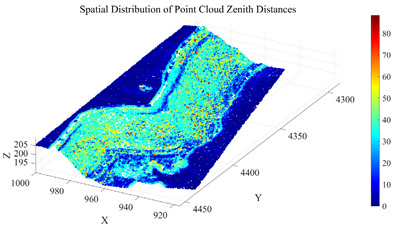	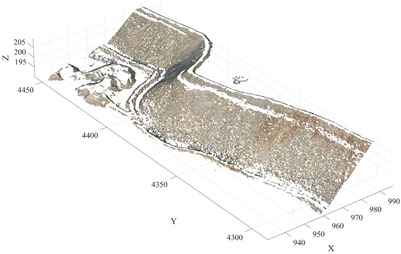	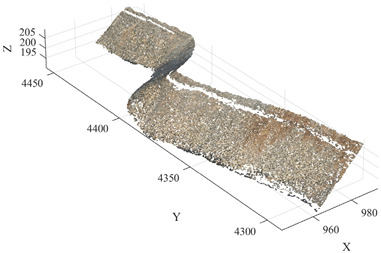	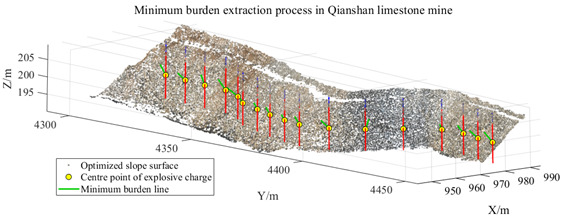
Qidashan, Anshan	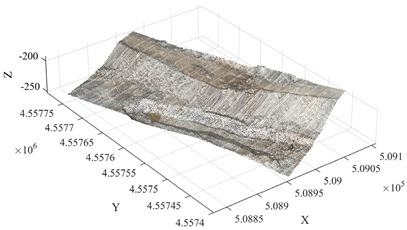	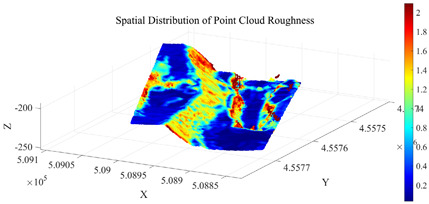	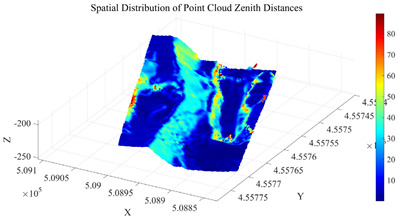	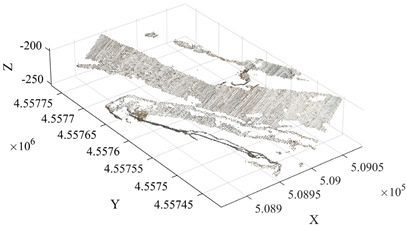	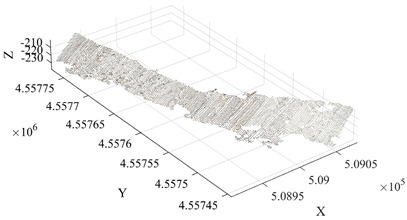	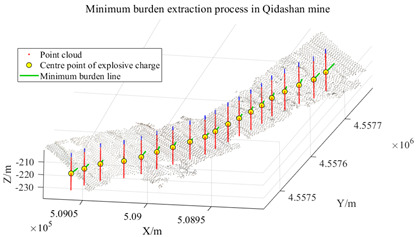

**Table 5:** Comparison of minimum burden extraction results under different schemes.

	Qianshan Limestone Open-Pit Mine (Liaoyang)							Qidashan Iron Open-Pit Mine (Anshan)						
Blast Hole No.	Measured Value(m)	Calculated Value			Absolute Error			Measured Value(m)	Calculated Value			Absolute Error		
		A	B	C	A	B	C		A	B	C	A	B	C
1	3.24	3.31	3.31	3.31	0.07	0.07	0.07	4.12	4.2	4.2	4.2	0.08	0.08	0.08
2	2.86	2.91	2.91	2.91	0.05	0.05	0.05	3.65	3.71	3.71	3.71	0.06	0.06	0.06
3	3.11	3.19	3.18	3.17	0.08	0.07	0.06	3.98	4.06	4.05	4.04	0.08	0.07	0.06
4	2.73	2.78	2.78	2.78	0.05	0.05	0.05	4.25	4.32	4.32	4.32	0.07	0.07	0.07
5	3.45	3.53	3.53	3.53	0.08	0.08	0.08	3.71	4.39	3.83	3.80	0.68	0.12	0.09
6	2.92	3.51	2.99	2.97	0.59	0.07	0.05	4.08	4.16	4.16	4.16	0.08	0.08	0.08
7	3.18	3.24	3.24	3.24	0.06	0.06	0.06	3.87	4.61	4.02	3.98	0.74	0.15	0.11
8	3.62	3.68	3.68	3.68	0.06	0.06	0.06	4.36	4.43	4.43	4.43	0.07	0.07	0.07
9	2.81	3.36	2.87	2.86	0.55	0.06	0.05	3.44	4.07	3.59	3.55	0.63	0.15	0.11
10	3.07	3.12	3.12	3.12	0.05	0.05	0.05	3.92	3.99	3.99	3.99	0.07	0.07	0.07
11	3.29	3.35	3.35	3.35	0.06	0.06	0.06	4.27	4.34	4.34	4.34	0.07	0.07	0.07
12	2.64	3.19	2.71	2.69	0.55	0.07	0.05	3.58	4.22	3.70	3.66	0.64	0.12	0.08
13	3.48	3.57	3.57	3.57	0.09	0.09	0.09	3.76	3.84	3.84	3.84	0.08	0.08	0.08
14	3.12	3.18	3.18	3.18	0.06	0.06	0.06	4.14	4.88	4.29	4.23	0.74	0.15	0.09
15	2.95	3.51	3.01	3.00	0.56	0.06	0.05	3.69	3.75	3.75	3.75	0.06	0.06	0.06
16	3.31	3.38	3.38	3.38	0.07	0.07	0.07	4.01	4.76	4.15	4.10	0.75	0.14	0.09
17	2.79	3.33	2.84	2.83	0.54	0.05	0.04	3.55	3.63	3.63	3.63	0.08	0.08	0.08
18	/							4.22	4.29	4.29	4.29	0.07	0.07	0.07
19								3.84	4.56	3.98	3.93	0.72	0.14	0.09
MAE	/	/	/	/	0.21	0.06	0.06	/	/	/	/	0.30	0.10	0.08

**Table 6:** Error statistics of different schemes at the Liaoyang Qianshan and Anshan Qidashan mines.

Mine	Scheme	Mean Absolute Error (m)	Standard Deviation of Absolute Error (m)	Maximum Absolute Error (m)	Mean Relative Error (%)	Standard Deviation of Relative Error (%)	Number of Blast Holes Exceeding 0.10 m
Liaoyang Qianshan	Scheme A	0.21	0.23	0.59	7.25	8.36	5
Liaoyang Qianshan	Scheme B	0.06	0.01	0.09	2.06	0.31	0
Liaoyang Qianshan	Scheme C	0.06	0.01	0.09	1.89	0.28	0
Anshan Qidashan	Scheme A	0.30	0.31	0.75	7.94	8.23	7
Anshan Qidashan	Scheme B	0.10	0.03	0.15	2.49	0.96	7
Anshan Qidashan	Scheme C	0.08	0.01	0.11	2.05	0.45	2

**Table 7:** Statistical Results of Preprocessing Error Propagation.

Error Source	Mean Error (m)	Maximum Error (m)	Impact on Minimum Burden
Octree downsampling	0.018	0.036	Mainly manifests as local smoothing, limited impact
Progressive morphological filtering	0.024	0.052	Mainly weakens local slope morphology
Boundary extraction deviation	0.021	0.047	Significant near slope crest and toe
Reference measurement uncertainty	0.018	0.018	Originates from RTK and laser scanner accuracy
Total propagated error	0.039	0.071	Smaller than the overall mean error of the proposed method

**Table 8:** Comparison of Minimum burden Extraction Results between the Improved DBSCAN Method and the Proposed Method.

Method	Number of Slope Points	Noise Point Proportion	Mean Absolute Error (m)	Mean Relative Error	Maximum Absolute Error (m)
Improved DBSCAN method by Liu et al. [16]	318,760	8.42%	0.141	4.73%	0.32
Proposed method	295,420	5.87%	0.077	2.68%	0.21

**Table 9:** Sensitivity Analysis Results of Key Parameters.

Parameter Type	Parameter Value	Mean Absolute Error (m)	Maximum Absolute Error (m)	Computation Time (s)	Result Description
Octree voxel size/m	0.04	0.075	0.20	312	More point cloud detail retained, but longer computation time
	0.06	0.073	0.20	246	Accuracy slightly improved, but increased computational cost
	0.08	0.077	0.21	185	Balanced accuracy and efficiency
	0.10	0.092	0.24	142	Local features begin to be smoothed
	0.12	0.118	0.29	116	Significant loss of slope detail
Roughness neighbourhood size K	10	0.112	0.31	176	Sensitive to local noise
	20	0.091	0.25	181	Noise influence reduced
	30	0.077	0.21	185	Balanced slope undulation and noise suppression
	40	0.086	0.23	191	Local structure slightly smoothed
	50	0.101	0.27	198	Insufficient detail representation
Neighbourhood coefficient c	1.5	0.124	0.34	168	Small neighbourhood, discontinuous slope surface
	2.0	0.096	0.26	176	Improved continuity
	2.5	0.077	0.21	185	Good balance between continuity and detail
	3.0	0.089	0.24	196	Boundary becomes blurred
	3.5	0.109	0.30	211	Non-target points increasingly included

## Data Availability

All data that support the findings of this study are included within the article.

## Abbreviations

The following abbreviations are used in this manuscript:

UAV	Unmanned Aerial Vehicle
DBSCAN	Density-Based Spatial Clustering of Applications with Noise
KNN	K-Nearest Neighbors
PMF	Progressive Morphological Filter
WCSS	Within-Cluster Sum of Squares
RTK	Real-Time Kinematic
GNSS	Global Navigation Satellite System
MAE	Mean Absolute Error
MRE	Mean Relative Error
LiDAR	Light Detection and Ranging
